# *LEPR c.668A>G* polymorphism in a cohort of Sri Lankan women with pre-eclampsia / pregnancy induced hypertension: a case control study

**DOI:** 10.1186/1756-0500-5-308

**Published:** 2012-06-19

**Authors:** Kamani Hemamala Tennekoon, Wijesekara Liyanage Indika, Rohan Sugathadasa, Eric Hamilton Karunanayake, Jayalath Kumarasiri, Ajita Wijesundera

**Affiliations:** 1 Institute of Biochemistry, Molecular Biology and Biotechnology, University of Colombo, No: 90, Cumaratunga Munidasa Mawatha, Colombo 3, Sri Lanka; 2 Castle Street Hospital for Women, Colombo 8, Sri Lanka

**Keywords:** Leptin, Leptin receptor polymorphism, Pregnancy induced hypertension, Pre-eclampsia

## Abstract

**Background:**

Leptin is known to be elevated in pre-eclampsia/ pregnancy induced hypertension (PE/PIH). However the reports on the association of leptin receptor *(LEPR) c.668A>G* polymorphism with PE/PIH are inconsistent.

**Findings:**

*LEPR c.668A>G* polymorphism was studied in a cohort of women with PE/PIH (N = 61) and normotensive pregnancies (N = 40) by polymerase chain reaction / restriction fragment length polymorphism. Genotype and allele frequencies were in Hardy-Weinberg equilibrium within both groups (Chi square test). Allele and genotype frequencies were not significantly different between PE/PIH and normotensive pregnancies (Chi square test). Leptin levels (Kruskal Wallis analysis of variance) and leptin/body mass index (one way analysis of variance) were not significantly different between genotypes within each group. However, leptin (Mann Whitney U test) and leptin normalised to body mass index (unpaired t test) were significantly higher in PE/PIH women homozygous and heterozygous for the G668 allele than in respective normotensives.

**Conclusions:**

Whether the leptin receptor *c.668A>G* polymorphism increases the risk of developing PE/PIH in Sri Lankan women remains inconclusive in view of the smaller sample studied. However leptin levels in PE/PIH appeared to be modulated by this polymorphism.

## Findings

### Background

Leptin, the first adipocyte hormone identified brings about biological effects via its cognate receptor [1]. Leptin receptor (LEPR) exists in several isoforms [2]. The full length isoform as well as some isoforms with short cytoplasmic domains are implicated in signal transduction [3-5]. LEPR isoform without the cytoplasmic and transmembrane domains is shed from the cells and circulates as the soluble leptin receptor. This modulates biological availability of leptin [6]. Several single nucleotide polymorphisms (SNPs) have also been identified in LEPR [7]. Of the SNPs reported, *c.668A>G* polymorphism in exon 6 (rs 1137101) has been reported to be associated with body mass index, breast cancer and age at menarche etc [8-10].

Pre-eclampsia / pregnancy induced hypertension (PE/PIH) is a pregnancy complication with increased fetal and maternal morbidity and mortality. It is thought to arise due to abnormal placentation followed by a maternal systemic disorder. Several studies including ours have shown that PE/PIH is associated with elevated circulating leptin levels and reduced levels of soluble leptin receptor compared to normotensive pregnancies [11-14]. We previously demonstrated that a polymorphism in the leptin gene increases risk of PE/PIH [14]. Studies on the association of *LEPR c.668A>G* polymorphism with PE/PIH are limited and findings have been inconsistent [15,16]. There are no data on the possible association of this polymorphism with PE/PIH for Sri Lanka or for any other South Asian population to the best of our knowledge. Thus we examined the possible association of *LEPR c.668A>G* polymorphism in a cohort of Sri Lankan women with PE/PIH.

### Results and discussion

There were 33 PE and 28 PIH patients in the PE/PIH group. Genotype and allele distribution was not significantly different between PE and PIH (Chi square test, P = 0.969; P = 0.964 respectively). Therefore both PE and PIH patients were analysed together. Both the PE/PIH and control group were predominantly Sinhalese with only 4 patients in the PE/PIH group and one subject in the control group belonging to other ethnic groups (ie: PE/PIH: 3 Muslims, 1 Tamil; control: 1 Muslim). Thus ethnicity was not taken into account in the analysis.

Genotype and allele distributions in PE/PIH and normal pregnancy are shown in Table 1. Heterozygosity for the *LEPR c.668A>G* polymorphism was the most common genotype in both groups. Genotype distributions were in Hardy-Weinberg equilibrium within PE/PIH (P = 0.752) and normotensive pregnancy (P = 0.710) groups. Furthermore genotype frequencies did not significantly differ between the two groups (P = 0.311). Though the *A668* allele was more frequent in PE/PIH than in normal pregnancy, the difference was not statistically significant (Chi square test: P = 0.372) and the *A668* allele did not confer a significantly higher risk of developing PE/PIH [relative risk (95% confidence limits) 1.126 (0.899 to 1.408)].

**Table 1:** Genotype and allele frequencies in pre-eclampsia/ pregnancy induced hypertension (PE/PIH) and normal pregnancy

	**PE/PIH: N (%)**	**Normal pregnancy: N (%)**	**P value**
Genotype			
*A668* homozygous	11 (18%)	06 (15%)	
*A668G* heterozygous	42 (69%)	24 (60%)	P = 0.311
*G668* homozygous	08 (13%)	10 (25%)	
Allele			
*A668*	64 (52%)	36 (45%)	
*G668*	58 (48%)	44 (55%)	P = 0.372

P values are for the Chi square test comparing PE/PIH and normal pregnancy.

Leptin levels and leptin normalized to body mass index of PE/PIH subjects included in the present study and some of the controls have been presented elsewhere and we reported that both the leptin levels and leptin normalized to body mass index were significantly higher in PE/PIH [14]. Reanalysis by *LEPR c.668A>G* genotype did not show any significant effect of the genotype on leptin levels or leptin normalized to body mass index (Table 2) within either group, though there was a tendency for levels to increase from AA genotype to GG genotype within the PE/PIH group. Leptin and leptin normalized to body mass index remained significantly higher in PE/PIH women who were heterozygous (p = 0.0007, p = 0.0026 respectively) and homozygous for the *G668* allele (p = 0.0085, p = 0.0189 respectively) than in the respective controls, but were not significantly different between PE/PIH and controls homozygous for the *A668* allele. Furthermore, BMI was significantly higher in heterozygotes (p = 0.0093) in the PE/PIH group compared to respective controls. BMI was higher in the *G668* homozygotes (p = 0.0616) in the PE/PIH group, but this did not achieve a statistical significance.

**Table 2:** Leptin levels (geometric mean and 95% confidence levels) and leptin normalized to body mass index (mean + SD) in pre-eclampsia/ pregnancy induced hypertension (PE/PIH) and normal pregnancy by genotype

**Genotype**	**PE/PIH Leptin ng/mL**	**Normal pregnancy Leptin ng/mL**	**P value**	**PE/PIH Leptin/BMI**	**Normal pregnancy Leptin/BMI**	**P Value**
*A668*	56.47	43.04	P = 0.1193	2.265 + 0.797	1.668 + 0.155	P = 0.0934
homozygous	(43.55, 73.21)	(34.91, 53.05)				
*A668G*	62.51	42.41	P = 0.0007	2.334 + 0.772	1.775 + 0.527	P = 0.0026
heterozygous	(56.48, 69.19)	(34.79, 51.69)				
*G668*	73.56	45.29	P = 0.0085	2.553 + 0.857	1.764 + 0.384	P = 0.0189
homozygous	(58.45, 92.57)	(35.27, 58.17)				

P values are for Mann Whitney U test comparing PE/PIH with normal pregnancy.

Association of leptin and polymorphisms of the leptin gene with increased risk of PE/PIH has been already well documented [12-14]. However, there are only a very few reports on the association of *LEPR c.668A>G* polymorphism with PE/PIH and these have remained inconsistent. A study from Hungary showed that the *A668* allele was significantly associated with severe PE [15] while no such association was observed in the HELLP syndrome [17], perhaps signifying differences in the underlying pathology. A recent study from Germany did not find an association of *LEPR c.668A>G* polymorphism with either PE or HELLP syndrome [16].

We previously demonstrated significantly higher plasma leptin levels and free leptin index (leptin levels/ soluble leptin receptor levels) in women with PE/PIH [14]. An increased risk of the disease was also conferred by the *A* allele of −2548 *G/A* polymorphism of the leptin gene. In the present study *LEPR c.668A>G* polymorphism of the same women with PE/PIH was studied. However, the prevalence of A and G alleles as well as different genotypes was not significantly different between PE/PIH and normotensive pregnancies. Thus our results contrast with those of Rigo et al [15], but support recent findings of Wiedemann et al [16].

Some investigators have shown that *G668* homozygosity is associated with significantly higher levels of leptin when compared to *A668* homozygosity [18,19]. In a recent study on a multiethnic cohort comprising of African, African-American, African-Caribbean, Caucasian and Asian/other ethnic groups this effect was observed only in Caucasians [20]. In the present study leptin levels and leptin normalized to BMI appeared to increase from *A668* homozygosity to *G668* homozygosity with heterozygotes having intermediate levels in PE/PIH. The differences did not reach a statistical significance presumably due to the smaller numbers in wild type and mutant homozygous groups. Such an effect was not seen among the controls.

LEPR has two cytokine receptor homology domains known as CRH1 and CRH2. *c.668A>G* polymorphism is located in the CRH1 domain. It has been suggested that CRH2 domain is adequate for signal transduction via LEPR. When the functional significance of this polymorphism was studied using a mouse model it did not show an effect on leptin signaling [21]. Our inability to observe an association of LEPR *c.668A>G* polymorphism with PE/PIH in the present study may have resulted from this lack of an effect on leptin signaling.

Increased maternal leptin in pregnancy is contributed both from the placenta and the adipose tissue. Following the delivery, maternal leptin levels return to pre-pregnancy levels confirming that the placenta is the major source of maternal leptin during pregnancy [22-24]. Even when accounted for adipose tissue contribution by normalizing leptin levels to BMI, genotype associated effect remained within the PE/PIH group indicating that the *LEPR c.668A>G* polymorphism is likely to modulate leptin levels in PE/PIH. A larger cohort of patients needs to be studied to confirm or refute this finding as well as to examine the effect of *LEPR c.668A>G* on the risk of PE/PIH in Sri Lankan women as the present study is underpowered in view of the smaller number of subjects studied.

### Conclusions

Whether the leptin receptor *c.668A>G* polymorphism increases the risk of developing PE/PIH in Sri Lankan women thus remains inconclusive due to the limited power of the present study. The effect of leptin receptor *c.668A>G* polymorphism on the risk of developing PE/PIH and whether *LEPR c.668A>G* polymorphism is likely to significantly modulate leptin levels in PE/PIH need to be studied using a larger cohort of patients.

### Methods

#### Subjects

Sixty one women with PE/PIH were selected from those who participated in a previous study on the association of leptin with PE/PIH. Their characteristics have been described in detail elsewhere [14]. Forty women with normotensive pregnancies were included as controls. Of these 19 subjects were selected from the control subjects from our previous study on the basis of availability of DNA aliquots. Remaining 21 subjects were recruited from the same tertiary care hospital according to the previously used study protocol. A sample size of 40 subjects in each group has a 90% power at a significance level of 0.05 to detect a 0.35 increase in the prevalence of *G668* allele in the test group assuming a prevalence 0.3 for the control group. Ethical approval from the Research, Ethics and Higher Degrees Committee of the Institute of Biochemistry, Molecular Biology and Biotechnology and written informed consent from the study participants were obtained prior to the study. Women in both groups had naturally conceived singleton pregnancies. Exclusion criteria were diabetes mellitus, gestational diabetes, chronic hypertension, renal disease, polycystic ovarian syndrome, menstrual cycle disturbances, previous history of infertility, as well as family history of diabetes mellitus or chronic hypertension.

On admission to the study, maternal age, height, weight and blood pressure were recorded and a sample of urine tested for proteinuria. Criteria for diagnosing PE/PIH have been described before [14]. Persistent (6 or more hours apart) blood pressure of at least 140/90 mmHg arising after 20 weeks of gestation was considered as PIH. Urine protein concentration >300 mg/l or more (or 1+ on a urine dipstick) in addition to hypertension was considered as pre-eclampsia. A peripheral venous blood sample was collected on diagnosis and before commencement of any treatment from the PE/PIH patients and on admission to the study in the third trimester from the controls.

Genomic DNA extracted from peripheral blood leukocytes was used for amplification by polymerase chain reaction (PCR) using exon specific primers. PCR products were digested with *Msp1* enzyme for restriction fragment length polymorphism analysis by Agarose gel electrophoresis [7].

Polymorphism at nucleotide position 668 from A > G changes codon 223 from CAG [coding for glutamine (Q)] to CGG [coding for Arginine (R)]. This change results in an *Msp 1* restriction site that cleaves the PCR product on exposure to the enzyme. Gels were manually scored as presence (*G668* homozygosity), absence (*A668* homozygosity) and heterozygosity of polymorphism. RFLP results were further confirmed by repeat analysis and by direct sequencing of representative samples. Chi-square test was used to ascertain whether the genotype and allele distributions were in Hardy-Weinberg equilibrium within each group, as well as to test whether the allele or genotype distributions differed between PE/PIH and normotensive pregnancies.

Leptin levels and leptin normalized to body mass index were compared between genotypes within each group using Kruskall Wallis ANOVA and one way ANOVA respectively. Leptin levels and leptin normalized to body mass index between PE/PIH and controls were compared after stratifying data by genotype using Mann Whitney U test and unpaired Student’s t test respectively.

## Competing interests

Authors declare that they have no competing interests.

## Authors’ contributions

KHT designed the study, analysed data and wrote the draft, WLI carried out genotyping and analysed data, RS collected samples and carried out leptin assays, EHK designed the study, JK and AW carried out clinical assessment of the subjects, all authors approved the final version of the paper.
